# The Relationship between Cadmium Toxicity and the Modulation of Epigenetic Traits in Plants

**DOI:** 10.3390/ijms22137046

**Published:** 2021-06-30

**Authors:** Lee-Ann Niekerk, Mogamat Fahiem Carelse, Olalekan Olanrewaju Bakare, Vuyo Mavumengwana, Marshall Keyster, Arun Gokul

**Affiliations:** 1 Environmental Biotechnology Laboratory, Department of Biotechnology, University of the Western Cape, Bellville 7535, South Africa; 3255882@myuwc.ac.za (L.-A.N.); 3341863@myuwc.ac.za (M.F.C.); 3779970@myuwc.ac.za (O.O.B.); 2 DST-NRF Centre of Excellence for Biomedical Tuberculosis Research, South African Medical Research Council Centre for Tuberculosis Research, Division of Molecular Biology and Human Genetics, Faculty of Medicine and Health Sciences, Tygerberg Campus, Stellenbosch University, Cape Town 7505, South Africa; vuyom@sun.ac.za; 3 Department of Plant Sciences, Qwaqwa Campus, University of the Free State, Phuthadithjaba 9866, South Africa

**Keywords:** DNA methylation, cadmium, chromatin, gene expression and histone acetylation

## Abstract

Elevated concentrations of heavy metals such as cadmium (Cd) have a negative impact on staple crop production due to their ability to elicit cytotoxic and genotoxic effects on plants. In order to understand the relationship between Cd stress and plants in an effort to improve Cd tolerance, studies have identified genetic mechanisms which could be important for conferring stress tolerance. In recent years epigenetic studies have garnered much attention and hold great potential in both improving the understanding of Cd stress in plants as well as revealing candidate mechanisms for future work. This review describes some of the main epigenetic mechanisms involved in Cd stress responses. We summarize recent literature and data pertaining to chromatin remodeling, DNA methylation, histone acetylation and miRNAs in order to understand the role these epigenetic traits play in cadmium tolerance. The review aims to provide the framework for future studies where these epigenetic traits may be used in plant breeding and molecular studies in order to improve Cd tolerance.

## 1. Introduction

The relationship between plants and their environment is highly complex. Plants are exposed to a multitude of stresses on a daily basis and therefore they have developed various strategies to combat these stresses [1]. These strategies include altering one or more of the following factors: morphological, biochemical and molecular factors [2]. One of the molecular factors that has been receiving increased attention in plants is the alteration of epigenetic factors [3]. It has been documented that epigenetic changes can be affected by both biotic and abiotic stresses [4,5].

Cadmium (Cd) is a toxic heavy metal which has detrimental effects on the environment which include the deterioration of soil quality, a decrease in plant health and a reduction in plant growth [6,7,8]. Due to mining, the use of chemical pesticides, improper watering practices and the non-ferrous metallurgy, the environment has become increasingly polluted with heavy metals such as Cd [9,10]. Due to Cd being nonbiodegradable, its high water solubility and its ability to change plant mineral uptake, it is often termed one of the most hazardous contaminants of soil [9,11]. To cope with adverse conditions such as high Cd concentrations some plants have evolved protective mechanisms such as the prevention of Cd entry in the roots, immobilization of Cd in vacuoles and the production of Cd phytochelatins [12,13,14]. These processes are dependent on the gene expression of certain genes and thus are controlled by genetic programming. DNA methylation is one of the more common epigenetic traits and it has been found to be important as it is involved in processes such as the induction of phenotypic alterations and maintaining genome stability when plants are under abiotic stress [15,16]. At times these alterations have been observed to improve the tolerance of the plants to specific abiotic stresses. Another important epigenetic trait includes chromatin remodeling, which has been reported to be active in many organisms when they are under stress. Certain authors have theorized that chromatin remodeling may be part of the mechanisms for stress memory [17]. Another important epigenetic trait, histone acetylation, has been shown to regulate and improve abiotic stress tolerance, such as salt tolerance in *T. aestivum*. The literature has displayed the importance of epigenetic modification and its role in the complex rearrangement of gene expression and the role it plays in crucial biological processes related to the plant’s response to environmental factors. To date, only a few studies have focused on the relationship between Cd and epigenetic changes in plants [18]. Therefore, this review describes the different epigenetic mechanisms which are involved in crop plants’ responses to Cd toxicity.

## 2. Plant Epigenetic Mechanisms for Cadmium Tolerance

### 2.1. Chromatin Remodeling and the Effect of Cadmium Stress in Plants

Chromatin is the repeating unit of nucleosomes, and it is composed of a complex of DNA and proteins assembled within the nucleus of cells [19]. Chromatin formation is carried out by a tightly condensed DNA wrapped around nuclear proteins called histones [20]. One hundred and forty-six base pairs of double-stranded DNA wrapped around eight histone proteins in a repeating unit are termed nucleosomes [21]. The more condensed a chromatin is, the harder it is for transcription factors and DNA-binding proteins to access DNA to carry out their functions [22]. Chromatin remodeling, therefore, is the rearrangement of chromatin from a condensed state to a transcriptionally accessible form, allowing transcription factors or other DNA binding proteins to access DNA and control gene expression [23]. Chromatin that is tightly packed and not actively being transcribed is referred to as heterochromatin. When the chromatin is packed loosely to allow transcription to occur, it is called euchromatin. Chromatin remodeling is highly implicated in the epigenetic modification of histone proteins such as demethylation/methylation, acetylation/deacetylation, which can change chromatin structure, causing transcriptional activation or repression [24].

This section’s focal point is to enumerate the effect of Cd on epigenetic modulation using chromatin remodeling. Understanding Cd utilization during chromatin remodeling involves two significant pathways which regulate DNA replication and nucleosome stability of chromatin structure [25]. One of these pathways is dependent on the histone-gene repressor (HIRA), and the other requires chromatin assembly factor 1 (CAF-1), which can be altered in response to Cd stress [26]. The variation of metals’ chemical properties and their reactive toxicities indicates that a uniform mechanism of action for all toxic metals is unlikely [27]. Heavy metals, including Cd, exert their cytotoxic and genotoxic effects by disrupting histone and other protein structures and functions by attacking the thiol groups in them, thus causing conformational changes to these proteins [28]. The overall effect of these conformational changes is the inhibition of DNA replication, gene expression, and cell division.

Few studies have investigated the effects of Cd on chromatin remodeling epigenetic modification in plants through the expression of stress-responsive genes. However, it is established that during Cd stress in plants, various forms of chromatin modification are possible, which include acetylation, methylation, phosphorylation, and ubiquitination that affect gene expression by altering chromatin structure and accessibility of transcription factors [29]. For example, histone’s hyperacetylation is generally associated with transcriptionally active chromatin, whereas deacetylated histone is correlated with inactive chromatin regions [30]. The heterotrimeric CAF-1 chaperone complex in *A. thaliana*, for instance, targets acetylated histone H3/H4 onto nascent DNA strand for the de novo assembly of nucleosomes [31]. Shafiq et al. [32] studied the interplay and coordination of histone acetylation and DNA methylation using metal stress tolerance dynamics as the focal point in *Z. mays*. The research found that Zn, Cd, and Pb differentially regulated DNA methyltransferase expression with varied histone deacetylases expression and dissuaded the excessive use of zinc fertilizers.

In another study by Gadhia et al. [33], the mitotic effects of inherited histone modification pathways in mouse embryonic stem cells (MES cells) were investigated, and the authors found that Cd exerted differential toxicity through selective disruption of chromatin on MES cells by targeting total histone protein (THP) production early in stem cell development with H3K27-monomethylation (H3K27me(1)) in later stages of differentiation. However, such a model study has not been replicated in plants and such a knowledge gap demands exploration by crop scientists and plant biotechnologists for future perspectives. Additionally, Zabka et al. [34] used apical root meristem in *V. faba* seedlings exposed to CdCl_2_ to monitor the epigenetic changes in transcription nucleosome assembly during the S-phase of the cell cycle. The study revealed cellular responses to Cd toxicity are interlinked with biochemical reactions through the generation of ROS and DNA-damage-induced replication stress.

### 2.2. The Role of DNA Methylation during Cadmium Stress

DNA methylation is a process involving the transfer of a methyl group to the C-5 position of the nucleotide cytosine and is an epigenetic modification which affects nuclear gene expression and genome stability [35]. DNA methylation/demethylation is a reversible reaction which allows this epigenetic trait to be associated with many diverse biological functions [18]. DNA methylation has been observed in all major taxonomic groups, which include mosses, ferns and angiosperms [36]. Given the functions that DNA methylation is involved in, as well as its conservation, it is quite evident that is required for proper plant development. DNA methylation of promoter regions has been shown to have an impact on gene expression and therefore affect the development of plants [37]. A study by Shafiq et al. [7] observed the effect Cd had on the DNA methylation levels in *T. aestivum* and how it affected metal tolerance. In the study the authors observed two *T. aestivum* cultivars, namely cv. Pirsabak 2004 (resistant) and cv. Fakhar-e-sarhad (sensitive), and tried to elucidate the mechanisms which could account for the metal tolerance observed in cv. Pirsabak 2004. The sensitive cultivar, cv. Fakhar-e-sarhad, was observed to have a higher DNA methylation level at the promoter regions of the transporters TaABCCs and TaHMA2 when compared to the resistant cultivar, cv. Pirsabak 2004, once exposed to Cd. It should be noted that TaABCCs and TaHMA2 are transporters which are required for metal transport [38,39]. The proposed theory was that the different levels of DNA methylation at the promoter sequences were due to Cd toxicity and resulted in altered expression of TaABCCs and TaHMA2 in the two cultivars and thus a difference in their metal transport capabilities was observed. A study by Fan et al. [9] observed the effect of inhibiting DNA demethylation on plant tolerance to Cd toxicity and iron nutrition. Cd exposure resulted in an increase in DNA methylation which was consistent with what was observed in the *T. aestivum* cultivar cv. Fakhar-e-sarhad in the study by Shafiq et al. [7,9]. The DNA methylation levels in Cd treated plants were 15% higher when compared to the control plants. The authors hypothesized that the demethylase genes (ROS1, DML2 and DML3) were important in the Cd-induced DNA methylation changes. This was investigated by using the *A. thaliana* rdd (ROS1/DML2/DML3) triple mutant. Rdd mutants exposed to Cd concentrations exceeding 20 µM displayed increased growth parameters when compared to the controls. This result confirmed that rdd mutants were more tolerant to Cd stress: the authors suggested that the improvement in tolerance was due to elevated DNA methylation in rdd mutants coupled with improved iron nutrition. The authors finally suggested that by inhibiting the RDD DNA, demethylation in the roots, which led to increased Cd tolerance, could be conferred through increasing DNA methylation and improving iron nutrition through a feedback mechanism. From the studies discussed it can be seen that epigenetic changes such as DNA methylation in plants when exposed to Cd are a complex system and may be different for different species of plants (Table 1). More studies are required to fully grasp the processes in which DNA methylation play a pivotal role, in order to improve plants against abiotic stresses such as Cd toxicity.

### 2.3. The Effects of Cadmium on Histone Acetylation

Histone acetylation was one of the earliest studied epigenetic mechanisms of transcriptional regulation. Histone acetylation is known to be involved in numerous and diverse cellular processes, including cell-cycle progression, DNA repair, gene silencing, growth and development, flowering, and seed development, and to deal with biotic stress and abiotic stress including salt, cold, and drought stresses [44]. Histone acetylation and deacetylation is a dynamic, reversible process catalyzed by two classes of enzymes, histone acetyltransferase (HAT) and histone deacetylase (HDAC), at lysine residues along the histone tail [45]. In general, histone acetylation correlates with transcriptional activation, and histone deacetylation correlates with transcriptional repression. Emerging evidence revealed that plant HATs and HDACs play essential roles in the regulation of gene expression in plant development and plant responses to environmental stresses. Furthermore, HATs and HDACs were shown to interact with various chromatin-remodeling factors and transcription factors involved in the transcriptional regulation of multiple developmental processes [46].

A recent study by Shafiq et al. [32] investigated the interplay between Zn, Pb and Cd for their uptake and translocation in *Z. mays*. In the study the authors observed that in the presence of Zn facilitates the accumulation and transport of Pb and Cd in the aerial parts of the maize plants. Moreover, the Zn, Pb, and Cd interplay specifically interferes with the uptake and translocation of other divalent metals, such as calcium and magnesium. Zn, Pb, and Cd, individually and in combination, differentially regulate the expression of DNA methyltransferases, thus altering the DNA methylation levels at the promoter of Zinc-regulated transporters, Iron-regulated transporterlike Protein (ZIP) genes to regulate their expression. Furthermore, the expression of HDACs varies greatly in response to individual and combined metals, and HDACs’ expression showed a negative correlation with ZIP transporters. Moreover, the Pb, Cd and Zn caused different histone acetylation profiles. Interestingly, their Pearson correlation analysis showed a negative correlation between the expression of HDACs/DNA methyltransferases and the expression of IRT1, ZIP1, ZIP3, ZIP5, ZIP6, and ZIP7, suggesting that the altered DNA methylation and acetylation levels, alone or together, could regulate the ZIPs’ gene expression. However, the expression of ZIP2 and ZIP8 showed a positive correlation with the expression of HDACs and DNA methyltransferases, suggesting that the regulation of these ZIP transporters could be independent of HDACs and DNA methyltransferases. However, further studies are required to validate the crosstalk between histone acetylation and DNA methylation to regulate the ZIPs’ gene expression. Their study highlights the implication of DNA methylation and histone acetylation in regulating the metal stress tolerance dynamics through Zn transporters and warns against the excessive use of Zn fertilizers in Cd contaminated soils.

In 2017, Lee and Back [47] investigated the overexpression of OsSNAT1 in transgenic *O. sativa*. In the pharmaceutical industry Ovine SNAT is utilized over plant SNAT for the overproduction of melatonin in plants due to the higher catalytic activity of animal SNAT. In this study, Lee and Back [47] overexpressed *O. sativa* serotonin N-acetyltransferase 1 (SNAT1), which is naturally expressed in chloroplasts, in *O. sativa*. In contrast to transgenic *O. sativa* overexpressing ovine SNAT, OsSNAT1-overexpressing transgenic *O. sativa* plants with elevated levels of melatonin showed significant tolerance to Cd and senescence, phenotypes that were not observed in transgenic *O. sativa* plants overexpressing ovine SNAT. Further studies revealed that homozygous SNAT1-overexpressing transgenic *O. sativa* plants had higher grain yields than wild-type controls under field conditions. Their data suggest that plant SNAT gene overexpression provides an opportunity to uncover new functions of melatonin that were previously unrecognized from studies in transgenic plants expressing animal SNAT genes.

Zabka et al. [34], explored the possible relationships and linkages between Cd (II)-induced oxidative stress and the consequent damage at the genomic level of in the *V. faba* seedlings exposed to Cd treatment and to post stress recovery water incubations. Exposure to Cd toxicity therefore leads to two types of consequent Cd-induced secondary stress conditions, including oxidative stress, by which reactive oxygen species (ROS) played an important part in producing disorder at the genomic DNA level eventually leading to replication stress. The phosphorylation of histone H2AX on Ser-139 (γ-H2AX) is regarded as one of the earliest responses involved in cellular perception/signaling pathways activated by DNA double-strand breaks [48]. By using immunofluorescence labeling of γ-H2AX as a highly specific and sensitive molecular marker for monitoring oxidative stress associated DNA damage, they observed an increase in the number of visible γ-phosphorylation of histone H2AX (serine 139) in root apical meristem cells of *V. faba* exposed to Cd and phosphorylation of histone H3 (serine 10). Furthermore, Cd^2+^ ions induced considerable changes in cell-cycle-related acetylation of H4 histone at the N-terminal K5 residue, whereas earlier studies concentrated mainly on the rDNA genes in *V. faba*, and suggested that H4 acetylation is loosely associated with their transcriptional activity and inversely correlated with nucleolar rDNA replication during the early stages of the S-phase. In conclusion, cellular responses to Cd (II) toxicity give the impression of being composed of a series of interlinked biochemical reactions which via generation of ROS and DNA damage-induced replication stress, ultimately activate signal factors engaged in cell cycle control pathways, DNA repair systems, and epigenetic adaptations.

### 2.4. The Relationship between Cadmium Tolerance and microRNAs

In plants, there are small non-coding RNAs known as microRNAs (miRNAs), which serve the purpose of base-pairing to their specific target mRNA, resulting in the initiation of silencing or degradation of mRNA translation [38,49]. In addition, miRNAs are known epigenetic modulators which affect protein levels of the target mRNAs without modifying gene sequences. MicroRNA-guided gene regulation at the post-transcriptional level is one of the molecular mechanisms that respond to heavy metal stresses [49]. These roles are well established; however, limited knowledge exists about their response to Cd and whether they are involved in Cd reduction in plants. Previous studies revealed that heavy metal-related gene expression was regulated by an arrangement of miRNAs [50,51]. The following studies investigated the molecular mechanisms of miRNAs in Cd tolerance in various plants.

A study conducted by He et al. [52] investigated the molecular mechanisms of Cd tolerance in *N. tabacum* plants. Applying miRNA high-throughput sequencing technology, He et al. [52] conducted a genomewide identification and analysis of miRNA in the roots of two *N. tabacum* cultivars (Guiyan 1 (sensitive) and Yunyan 2 (resistant)). The authors employed qRT-PCR to validate the findings of the high-throughput sequencing, and obtained the validated miRNA profiles: they then utilized the miRBase database to classify tobacco miRNAs. He et al. [52] utilized RNAfold and Mireap to obtain novel miRNAs. Using the tobacco DFC gene index and psRNA target tool, the authors calculated putative targets. Cd stress caused significant alteration in miRNA length regardless of cultivar variation [52]. The authors suggested that alteration implicates miRNA involvement in extensive regulation of gene expression in response to the Cd stress in the *N. tabacum* roots. They found 72 and 14 differentially expressed miRNA, known and novel (respectively), of which 28 of the known and 5 of the novel miRNAs were deemed as Cd tolerance associated miRNA. Cadmium concentrations were then analyzed within plant organs, and it was noticed that the concentration was higher within the roots and shoots of both cultivars compared to their respective controls.

During the study it was recorded that Yunyan 2 accumulated more cadmium (exhibited higher cadmium concentration in both roots and shoots organs) than Guiyan 1 under cadmium conditions; however, Yunyan 2 still exhibited higher cadmium tolerance when compared to Guiyan 1. Yunyan 2 was considered less sensitive to cadmium due to only indicating a 16.19% and 54.44% reduction in plant height and SPAD value (measures leaf chlorophyll concentration), respectively, when compared to its control. Guiyan 1 cultivar, on the other hand, displayed a 27.75% and 60.22% reduction in plant height and SPAD value, respectively, when compared to its control. Zhou et al., 2019 ultimately graded each with cultivar an integrated score of 43.173 (Guiyan 1) and 35.077 (Yunyan 2).

The study then provided the addition of new novel mRNA to the tobacco microRnome, and He et al. [52] concluded that 28 known and 5 novel miRNAs were linked to Cd tolerance in Yunyan 2 cultivar. The Cd tolerance displayed by Yunyan 2 was suggested to be due to the miRNA profile, indicating repression of certain miRNAs that accounted for efficient scavenging of Cd-induced ROS, thereby reducing Cd toxicity in tobacco. The profile also indicated that the regulation of processes involved in cell growth, ion homeostasis, stress defense, redox maintenance and hormone signaling contributed to better growth performance in Yunyan 2 than in the Guiyan 1 cultivar.

The plant miRNA miR390 is extremely vital in Cd stress responses [49] and this miRNA is conserved in a few plants (*O. sativa*, *A. thaliana* and *Z. mays*). In the *O. sativa* genome, miR390 is encoded to regulate the expression of the *O. sativa* stress-responsive leucine-rich repeat receptorlike kinase by cleaving the *OsSRK* mRNA [49].

**Figure 1:**
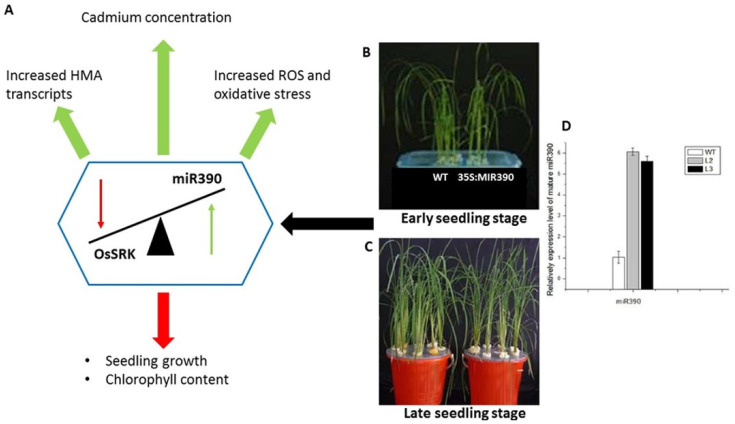
Adapted from Ding et al., 2016. Represents the transgenic *O. sativa* (35S:MIR390) and the effects of the overexpression of miR390 under cadmium stress. (**A**) Indicates how the overexpression influenced the OsSRK expression and how this expression relationship altered the biochemistry of *O. sativa* seedlings. (**B**) Represents the physiology of the Wild type (WT) and transgenic *O. sativa* plant in their early seedling stage and (**C**) represents the physiology of the Wild type (WT) and transgenic *O. sativa* plants in their late seedling stage. (**D**) A graphical representation of the relative expression of miR390 in both WT and transgenic lines, confirming the overexpression of miR390. The green arrows indicate an increase and the red arrow indicates a decrease.

Ding et al. [49] employed a transgenic approach to investigate miRNAs role in the *O. sativa* response to Cd stress. *O. sativa* was transformed with a MiR390 overexpression plasmid, followed by validation of transgenic plants by analyzing their transcript levels of mature MiR390 and *OsSRK*. [49]. Transgenic plants exhibited a significant increase in miR390 transcripts (Figure 1), confirming overexpression and the *OsSRK* transcripts were much lower than the controls. Ding et al. [49] recorded an increase in *OsHMA2*, known to transport Zn/Cd from the roots to shoots, and *OsNRAMP5*, known to transport Mn and Cd into the roots in the transgenic plants under Cd stress [53,54]. The study recorded that in the presence of Cd the transgenic lines accumulated more Cd, which resulted in Cd toxicity. The conclusion was that these transcripts (*OsHMA2* and *OsNRAMP5*) were positively regulated by miR390, indicating their importance in Cd accumulation and translocation in *O. sativa* plants. The seedling growth of transgenic lines was retarded, the chlorophyll content decreased, and MDA and H_2_O_2_ increased by 40% and 2-folds, respectively, when compared to the control plants [49]. The outcomes of the study by Ding et al. [49] correlated with a study conducted by Marin et al. [55] which concluded that in response to Cd stress, miR390 exerted a negative effect on the lateral root formation of *A. thaliana* plants (regulated the pathway involved in auxin signaling and lateral root development). Considering all of the results obtained, it can be concluded that miR390 acts as a negative regulator involved in Cd tolerance in *O. sativa* plants and that the expression of miR390 alters the expression pattern of several metal transporter genes, resulting in Cd accumulation and toxicity in *O. sativa* plants.

**Figure 2:**
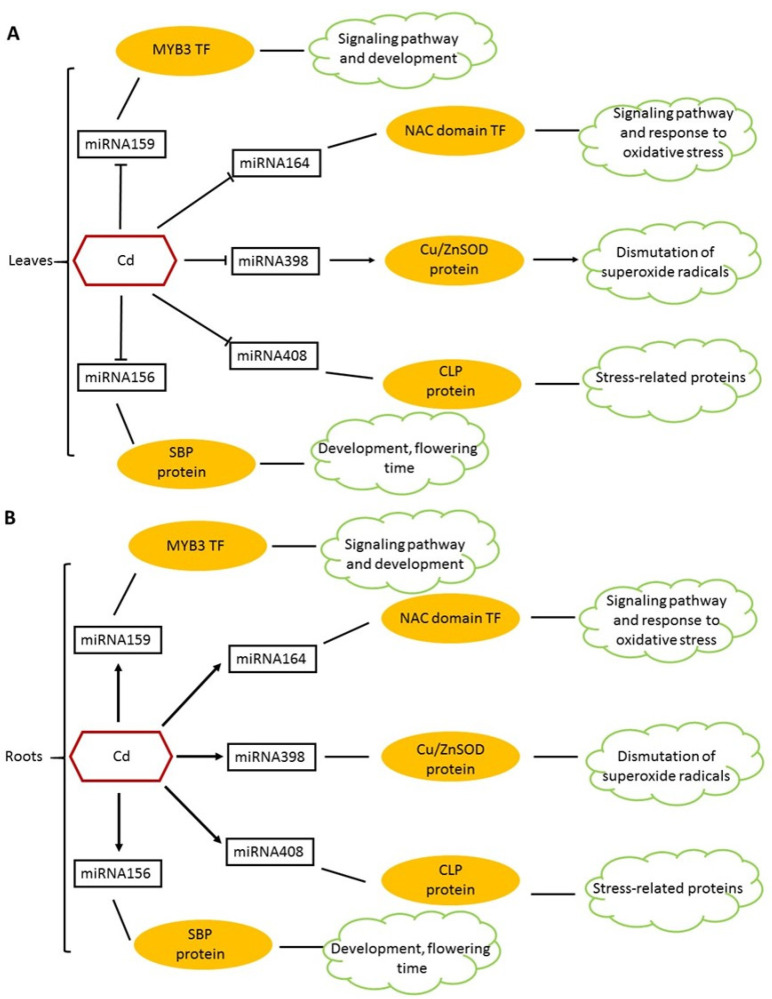
Represents the miRNA expression profiles in *T. aestivum* plants under CD stress. (**A**) Represents the 5 miRNAs that were downregulated in the leaves at 12 h after exposure to Cd stress. The miRNA159 (targets MYB3 transcription factor), miRNA164 (targets NAC domain Transcription factor), miRNA398 (targets CSD) and miRNA408 (targets CLP). Besides the other 4 miRNAs, miR156 remained downregulated at all time points within the study. (**B**) Represents the 5 miRNAs that were upregulated in the roots from 12 h until 24 h after exposure. miRNA408 remained upregulated at all time points in this study. The arrows indicate the expression of the different micro RNAs, yellow ovals represent the targets of the miRNAs and the green clouds represent the function of the proteins. Negative correlations in the transcript expression were experienced between miR398 and CSD in the roots, miR159 and MYB3 in the roots and leaves, miR408 and CLP in the roots and leaves and between miR164 and NAC in the leaves. There was a positive correlation between miR156 and SBP in the roots and leaves. Adapted from Qiu et al. [56].

**Figure 3:**
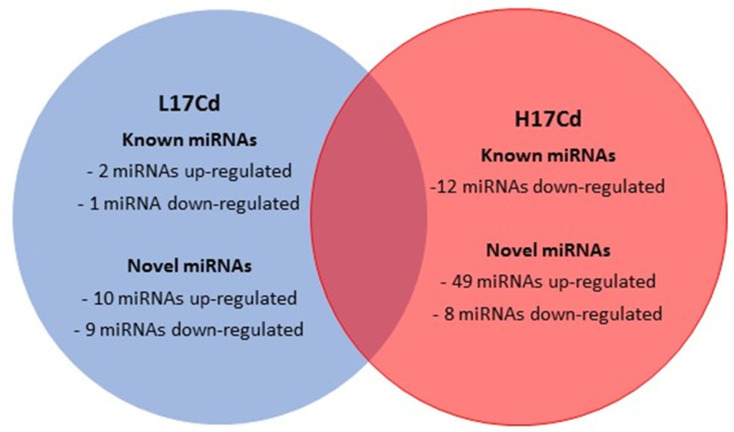
Adapted from Zhou et al., 2019. Represents a Venn diagram of the differential expression patterns (DEP) between *L17Cd* and H17Cd wheat cultivars, compared to their controls, under cadmium stress. The DEP was obtained by conducting next generation transcriptomic and miRNA sequencing. Zhou et al., 2019 noted that miR9664-3p and tea-miR159a (known positive role in *P. striiformis* infection) in seed were both upregulated in the *L17Cd* cultivar when compared to its control *L17CK*, however, these two miRNAs were downregulated in H17Cd.

In a study conducted by Qiu et al. [56], Real-Time PCR was utilized to obtain the expression profiles of *T. aestivum* microRNA. The webtool, psRNATarget, was employed to recognize the miRNA targets (mRNA). Qiu et al. [56] recorded different miRNA expression profiles under Cd stress, in the roots and shoots of *T. aestivum* seedlings. Physiological variations, such as reduction in shoot and root elongation, were observed between the control seedling and the Cd treated seedlings. The relative water content of the leaves of the Cd treated seedlings was reduced (67%) when compared to the control seedlings (94–96%) after 48 h of exposure to Cd. The chlorophyll content of the treated seedlings was reduced, and the lipid peroxidation of the membranes was increased under Cd stress [53]. Figure 2 exemplifies the five miRNAs differentially expressed between the roots and shoots of Cd treated seedings [56]. miR398 was observed to target CSD, a Cu/Zn Superoxide Dismutase (a ROS scavenger). A reduction in miR398 expression led to an increase in CSD expression [56]. However, this decreased expression in miR398 indirectly led to the increase in hydrogen peroxide production (correlates to the increase in lipid peroxidation) due to the role of CSD in the dismutation of superoxide radicals to produce more hydrogen peroxide [57]. Thus, miR398 was deemed a main constituent for the detoxification of ROS, by the control of the target gene, CDS [56]. To conclude, Qiu et al. [56] suggested wheat miRNAs are involved in the facilitation of Cd stress signaling responses and, furthermore, suggested that the differential expression patterns of miRNA observed in *T. aestivum* roots and leaves suggest that there are variations in the functional roles of miRNA in particular organs of *T. aestivum* plants with regard to the regulation of plant tolerance [53]. Another study by Zhou et al. [58] took a different approach in which they focused on the regulatory mechanisms between heavy metal ATPases (HMAs) and microRNAs in *T. aestivum* plants. The aim was to comprehend the global transcriptional response of *T. aestivum* plants to Cd stress and how this response was controlled by miRNAs. Two wheat cultivars (low-Cd accumulation (L17) and High-Cd accumulation (H17)) were analyzed using next generation transcriptomic and miRNA sequencing to the miRNA profiles of each cultivar in response to Cd stress. qRT-PCR was employed to obtain and observe the differentially expressed miRNA profiles and the TaHMA profiles in the root sections [58]. Figure 3 exemplifies the differential expression between the two Cd treated cultivars. The results obtained in this study noted two particular miRNAs (miR9664-3p and tea-miR159a) which were upregulated in *L17Cd* (Cd treated), compared to its control *L17CK*, but downregulated in H17Cd [58]. Further analysis demonstrated that the putative targets of both miR9664-3p and tea-miR159a were downregulated in *L17Cd* plants. Han et al. [59] previously recorded that tea-miR159a expression in leaf and developing seeds of wheat plants play a positive role in the response to *Puccinia striiformis*, through the regulation of TaMyb3 expression [59,60]. Zhou et al. [58] also identified 32 TaHMA genes in *T. aestivum* plants. Zhou et al. [58] deduced that miRNA can regulate TaHMAs; however, further validation was required for future works. Concluding, both studies [56,58] depict miRNAs as potentially playing an essential role in Cd responses in *T. aestivum* plants, either through the regulation of the miRNAs responsible in ROS scavengers or in the regulation of the TaHMA2;1 gene.

## 3. Conclusions

Knowledge about Cd tolerance in plants has been extensively documented and updated with regard to physiological and biochemical effects and responses to the toxic Cd conditions. An increase in Cd uptake in plant cells mainly leads to a decrease in plant growth, development and yield. In order to improve plant growth under high Cd concentrations, researchers identified various key genes and proteins which regulate Cd tolerance in various plants. In addition to these molecular mechanisms, epigenetic mechanisms have emerged as an important complex factor in plant responses to heavy metal stresses. However, very few studies have focused primarily on epigenetic mechanisms to improve plant performance under Cd stress. Epigenetic mechanisms could protect plants from Cd-induced DNA damage through random DNA methylation as well as through direct epigenetic regulation of Cd stress responsive genes. The interplay between chromatin remodeling, DNA methylation, histone modification, and microRNAs provides plants with a multilayered and robust epigenetic mechanism to improve survival under Cd stress. Therefore, there is a need to develop more robust bioinformatic pipelines for epigenetic analysis in plants under Cd stress. Furthermore, these studies will be crucial in future plant breeding studies where more stable epigenetic plant genotypes with improved Cd tolerance will be developed.

## Figures and Tables

**Table 1:** The relationship between Cd stress and DNA methylation in plants.

Plant	Treatment Doses and Duration	DNA Methylation Due to Cd	Notable Observation	Reference
*Oryza sativa*	80 µM for 4 days	Decreased DNA methylation	Promoted rice growth	[40]
*Posidonia oceanica*	10 µM and 50 µM for 4 days	Increase in DNA methylation (hypermethylation)	Apoptotic features of cells	[41]
*Zostera marina*	8.9 µM for 6 days	Increased DNA methylation	*ROS1* (DNA demethylation downregulated)	[42]
*Arabidopsis thaliana*	0.5 mg·L^−1^ for 15 days	Increased DNA methylation (Hypermethylation)	DNA methylation polymorphisms	[43]
